# Separation of α‑Terpineol and Limonene from an Orange Essential Oil Mixture Using Supercritical CO_2_ Pressure Reduction

**DOI:** 10.1021/acsomega.5c08436

**Published:** 2025-10-25

**Authors:** Rayanne Priscilla França de Melo, Rafael Chelala Moreira, Glaucia Maria Pastore, Juliano Lemos Bicas, Julian Martínez, Luana Cristina dos Santos

**Affiliations:** † School of Food Engineering, Department of Food Engineering and Technology, 28132 University of Campinas, 80, Monteiro Lobato Street, Campinas 13083-862, São Paulo, Brazil; ‡ School of Food Engineering, Department of Food Science and Nutrition, University of Campinas, 80, Monteiro Lobato Street, Campinas 13083-862, São Paulo, Brazil; § Food Science Research Institute (CIAL), CSIC-UAM, Nicolás Cabrera 9, Madrid 28049, Spain

## Abstract

Brazil is the world’s largest orange producer, generating significant amounts of byproducts that are used to produce limonene-rich orange essential oil. In this sense, one possible alternative for its valorization is the biotransformation of limonene into α-terpineol, which has emerged as a promising valorization route, but the efficient separation of these compounds remains challenging due to their similar chemical nature. This work aimed to evaluate the solubility and fractionation behavior of α-terpineol and limonene in supercritical CO_2_ (SC-CO_2_) using a model mixture (orange essential oil + α-terpineol, 60:40 wt %), which simulated a biotransformation product. The best solubilization was found at 10 MPa and 40 °C. Supercritical fluid fractionation (SFF) was performed at different separator pressures (6–8 MPa) and temperatures (40–60 °C), to find optimal conditions for the selective recovery of α-terpineol. The SFF performed at 8 MPa and 40 °C achieved the precipitation of most α-terpineol. However, coprecipitation of limonene indicated limited selectivity under the tested conditions, likely due to molecular interactions in the complex multicomponent matrix. Calculated losses through the SFF at this same condition indicated a substantial limonene loss of nearly 50%, highlighting the challenges in designing an efficient system for terpene separation.

## Graphical abstract

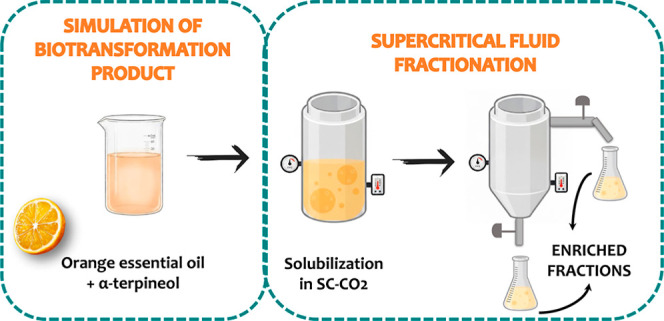

## 1 Introduction

The Brazilian orange production reached 17.6 million tons in 2023,^1^ making the country responsible for approximately one-third of the world’s production, which is destined for both juice processing and consumption in its fresh form. Consequently, residues such as peels and seeds are generated in large quantities. As an alternative to reduce these residues amounts, orange essential oil is currently obtained by cold pressing of seeds, pulp, and peel residues.^2^ Although the composition of orange essential oil can vary according to the type of orange, climate, or season, these oils are generally mixtures with a complex chemical composition in which limonene is the major compound, representing over 70% of the volatile fraction of the oil, followed by other terpenes with relevant application such as linalool, myrcene, carveol, and α-terpineol.^3^

Despite its lower concentration in comparison to limonene in the composition of orange essential oil, α-terpineol, a monoterpene alcohol, is a noble compound due to its remarkable sensory properties, which can be used as a flavoring ingredient in frozen foods, confectionery, and beverages.^4^ Moreover, some studies also report possible biological activities of α-terpineol, such as anticancer, anticonvulsant, antiulcer, antihypertensive, and antinociceptive agents.^5^ Therefore, some methods have been investigated for the production of α-terpineol through chemical synthesis^6^ or by biotransformation of limonene into α-terpineol using the orange essential oil as the substrate, where the latter was recently reported by Molina et al.^7^ achieving 182 g α-terpineol/L microorganism culture, a notable advance in comparison to the first results from Kraidman et al.,^8^ who reported only 1 g/L from the same biotransformation reaction.

Despite biotransformation being a promising procedure to concentrate compounds, fractionation processes using supercritical fluid technology can also assist as sustainable alternatives to concentrate α-terpineol. In this context, supercritical carbon dioxide (SC-CO_2_) is often chosen for essential oil fractionation, due to its nontoxic property and tunability, allowing for selectivity that ranges from gas-like to liquid-like properties, operating at relatively low pressures and near room temperature.^9^

Therefore, Supercritical Fluid Fractionation (SFF) using separators is of relevant importance in bioactive compounds concentration, since it explores the selectivity of CO_2_ by only modifying its temperature and pressure, thus promoting the separation of targeted molecules from mixtures by pressure reduction.^10–12^ SFF offers significant advantages over conventional separation techniques, since it is a continuous process that uses an inert, nontoxic, and recyclable solvent, in line with green chemistry principles and the reduction of environmental impact. The operation at moderate temperatures enables the preservation of thermolabile compounds, while the high selectivity ensures greater efficiency in complex separations. Furthermore, the modular nature of the technology facilitates industrial scalability and integration into diverse production chains with economic feasibility. In this context, SFF emerges as a sustainable and technically robust alternative, either as a primary process or combined with other separation techniques. For an efficient separation, it is important to determine the solubility of the target compounds in SC-CO_2_ in order to define the most suitable pressure and temperature conditions for SFF. The solubilities of compounds of orange essential oil have been previously reported, providing valuable data for evaluating prediction models that estimate their solubility in SC-CO_2_ ^13–15^ and thus assess the efficiency of the proposed fractionation processes.

The fractionation of compounds from essential oils presents a significant challenge due to their nature as multicomponent systems composed of highly complex mixtures. However, terpenes can be effectively separated from essential oil mixtures under the appropriate temperature and pressure conditions that enable selective fractionation. Given the importance of α-terpineol as a food ingredient and its potential pharmacological applications, it is noteworthy that, to the best of our knowledge, only limited studies have reported on the separation of α-terpineol and limonene from orange essential oil using SC-CO_2_.^16,17^ Therefore, this study aimed to elucidate the solubility and fractionation behavior of α-terpineol and limonene in SC-CO_2_, adding value to this food industry byproduct. To achieve this, a mixture of orange essential oil enriched with α-terpineol (designed to simulate a pseudo-binary representative system of a biotransformation product) was fed to a SFF system using CO_2_ as solvent. The effects of the temperature and pressure of the separator on the yield and composition of limonene and α-terpineol of the resulting fractions were investigated, finally obtaining the optimized conditions for a selective recovery of α-terpineol.

## 2 Materials and Methods

### 2.1 Simulation of the Biotransformation Product Enriched in α-Terpineol

First, in order to obtain a representative material, a model mixture was prepared prior to SFF. The composition of the mixture was based on previous results that maximized the biotransformation of limonene into α-terpineol, obtaining a mass proportion of precisely 59.3% of limonene and 40.7% of α-terpineol.^7^ Therefore, 60% of the essential oil (gently donated by Citrosuco, located in Matão, Brazil) was mixed with 40% of α-terpineol (96% purity, Sigma-Aldrich, Brazil) in order to simulate an approximate composition (wt %) of the biotransformed mixture (considering that the major compound in the essential oil is limonene).

### 2.2 Dynamic Solubility in Supercritical CO_2_

The SFF process performed in this work was based on the modification of pressure and temperature in a separator, carried out in order to precipitate part of the compounds initially dissolved in SC-CO_2_. Therefore, it was mandatory to guarantee that the compounds aimed to be separated would be completely dissolved in SC-CO_2_ prior to fractionation. In order to determine the solubility of the simulated mixture and their initial solutions (essential oil and pure α-terpineol) in supercritical CO_2_, the method described in dos Santos et al.^18^ was applied with some modifications. The pressure ranged from 8.5 to 20 MPa, while temperature was from 40 to 60 °C. First, approximately 10 g of the samples were soaked in a towel paper roll and placed into a 100 mL stainless steel solubilization vessel. CO_2_, initially stored in cylinders, flowed through a coil in a refrigeration bath (−10 °C) (Marconi MA-184, Piracicaba-SP, Brazil) to ensure its liquid state before being pumped up to the work pressures in a high-pressure pump for liquids (MAXIMATOR M-11 CO_2_, Nordhausen, Germany). Once the work pressure was achieved, CO_2_ was heated by a coil immersed in a heating bath (Marconi, MA-184, Piracicaba-SP) to reach the work temperature. The same bath heated a stainless-steel jacket that surrounds the solubilization vessel in order to keep the temperature constant throughout the process. After the process pressure and temperature were reached, a static time of 15 min was maintained under these conditions. Next, the micrometer valve was opened to start the flow of CO_2_ containing the compounds that was solubilized under a minimum flow rate of 1.8 g/min to avoid sample entrainment by the solvent. The system was then completely depressurized, and the material was collected in glass vials and weighed. In order to avoid possible volatilization of the collected compounds, the flasks were partially immersed in an ice bath. Moreover, cotton was placed inside the flasks as a trap system. The CO_2_ mass used for solubilization was calculated from the measured volume used during the process, accounted in a gas totalimeter (LAO, Osasco, Brazil), using the density from the National Institute of Standards and Technology (NIST) database.^19^ The solubility (Y*) of the separated solutions and their mixture in SC-CO_2_ was then calculated as the mass ratio between the collected fraction and total used CO_2_, following eq 1.

1 $$Y^{*}=\frac{mass\;of\;collected\;fraction}{mass\;of\;{CO}_{2}}$$

### 2.3 Supercritical Fluid Fractionation (SFF)

To achieve the fractionation of the mixture, pressure reduction was carried out in a separator vessel coupled to a solubilization cell, which was controlled by a back pressure regulator. The micrometer valve was placed between the solubilization unit and the separator, which allowed maintaining the CO_2_ flow rate at 8.6 g/min. The process flow diagram is depicted in Figure 1.

**Figure 1:**
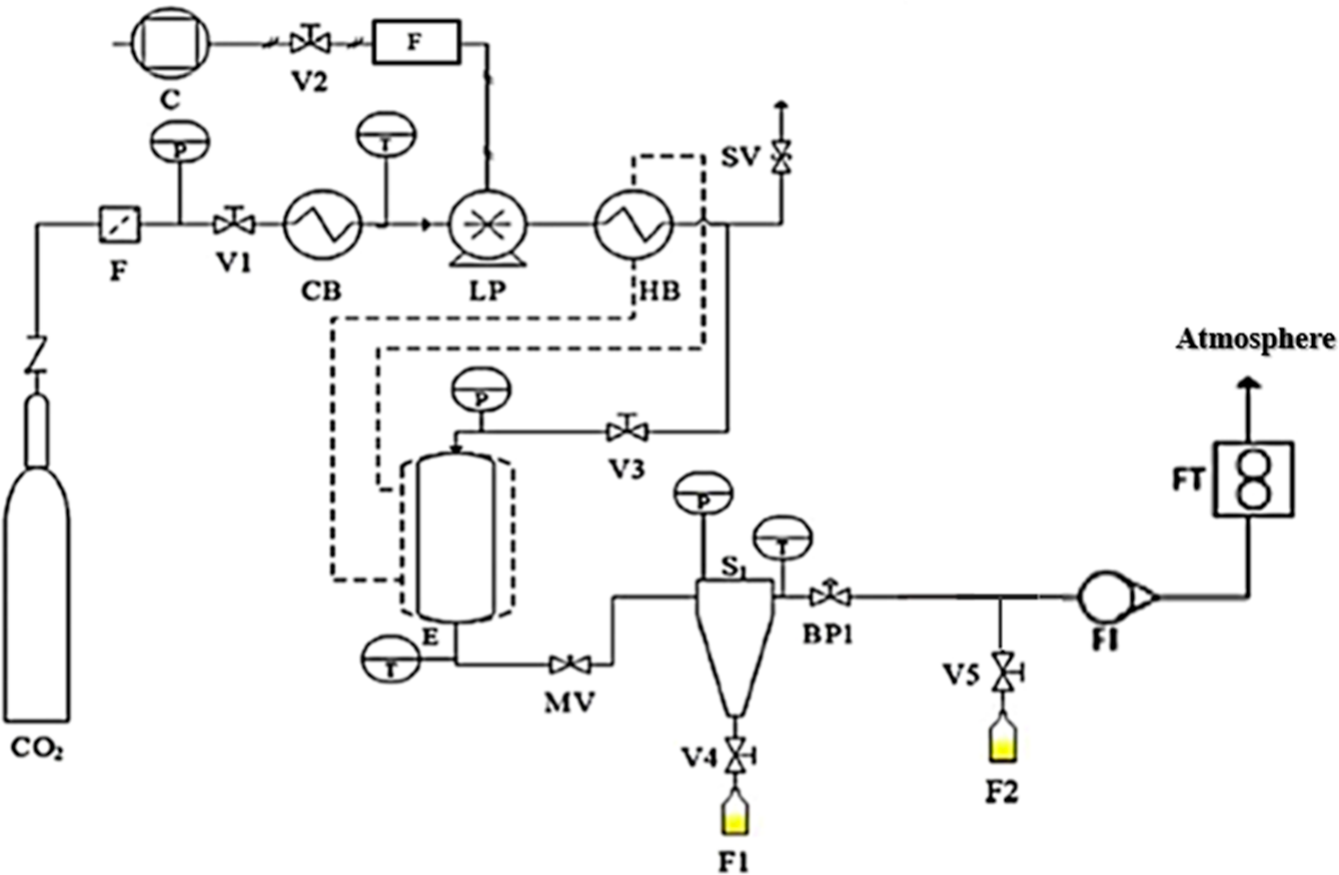
Flow diagram of the Supercritical Fluid Fractionation (SFF) unit: F—CO_2_ filter; T—temperature gauge; C—compressor; V1 to V5—on/off valves; MV—micrometering valve; SV—safety relief valve; BP1—back pressure regulator; CB—cooling bath; LP—liquid pump; HB—heating bath; E—solubilization vessel; S1—separator; F1—collection flask of the fraction precipitated in S1; F2—collection flask of the depressurized fraction; FI—CO_2_ flow indicator; FT—flow totalizer (in m^3^) (adapted from dos Santos et al.^10^).

#### 2.3.1 SFF Kinetics

A kinetic experiment was conducted to determine the solvent-to-feed mass ratio (S/F) for the SFF process. The S/F ratio is a key parameter for optimizing supercritical fluid operations and has been widely used in the literature by other authors.^20–22^ The SFF kinetics was performed under solubilization conditions of 10 MPa and 40 °C, which provided the highest solubility of the mixture of essential oil and α-terpineol (according to the method described in Section 2.2). 10 g of a mixture of orange essential oil and α-terpineol mixture (60:40 wt %) was placed into a 100 mL solubilization vessel. The system was brought to the working pressure and temperature (as described in Section 2.2), and once these conditions were reached, it was kept closed for a static period of 15 min. The separator was maintained at 7.5 MPa and 50 °C. These conditions were selected based on the fractionation of a pseudoternary system (linalool + limonene + CO_2_) studied by Cháfer et al.,^14^ who demonstrated that such settings allow for more efficient separation of a terpene (limonene) from an oxygenated terpene (linalool) using SC-CO_2_. Since the present mixture also contains an oxygenated terpene (α-terpineol), the same conditions were reproduced. Fractions precipitating in the separator were collected in glass flasks and weighed over a one hour period with a constant CO_2_ flow. The resulting data were used to construct an accumulated mass curve (kg extract/kg feed) as a function of time.

#### 2.3.2 Influence of Temperature and Pressure in SFF

For the fractionation process, experiments were conducted at pressures ranging from 6 to 8 MPa and temperatures between 40 and 60 °C in the separator, while the solubilization step was maintained at 10 MPa and 40 °C. It is important to note that, under certain conditions, the pressure was below the critical pressure of CO_2_ ( $P_{c}^{{CO}_{2}}$ = 7.34 MPa) meaning that CO_2_ was in the gaseous state. Nevertheless, the process is termed “supercritical fluid fractionation” for convenience. After solubilization, the mixture entered the separator where the pressure was reduced. Compounds with lower affinity for CO_2_ became less soluble and precipitated in the separator, forming fraction *F*1. In contrast, compounds that remained dissolved in CO_2_ under each condition were recovered by depressurization through a micrometer valve, yielding fraction *F*2.

SFF was carried out following the methodology proposed by dos Santos et al.,^10^ with some adaptations. As previously described in Section 2.2, the solubilization cell was loaded with 10 g of a mixture of orange essential oil and α-terpineol (60:40, wt %) and pressurized with SC-CO_2_ to 10.0 MPa and 40 °C (defined as the optimal solubilization condition). After a static period of 15 min, the micrometer valve was carefully opened until a stable flow rate of 8.6 g/min was achieved. The fraction of the mixture that became insoluble under the applied SFF conditions precipitated at the bottom of the separator vessel, while the soluble fraction was collected after complete depressurization at the end of the process, which ended when the defined S/F ratio was reached (Section 2.3.1). Both fractions were collected in glass flasks, weighed, and stored at −18 °C until further analyses.

### 2.4 Chemical Characterization

#### 2.4.1 Characterization by Gas Chromatography-Flame Ionization Detector (GC-FID)

In order to quantify limonene and α-terpineol present in the samples and, therefore, evaluate the efficiency of the SFF process, the compounds were identified by similarity of retention time of the respective standard and quantified with the aid of a calibration curve for the same compound. Thus, the GC-FID analyses were performed on (i) orange essential oil; (ii) the mixture of orange essential oil and α-terpineol (60:40 wt %); and (iii) the precipitated (*F*1) and depressurized (*F*2) fractions obtained in each SFF condition.

Although the GC-FID procedure yields concentrations in g/L, these values were subsequently converted (using the correspondent density) to absolute masses and recoveries (%), thereby facilitating the assessment of process efficiency on a mass basis. The samples were characterized following the methodology described in Bicas et al.^23^ Briefly, the separation was performed in an HP-6890 Plus gas chromatograph (Agilent, Santa Clara, CA-USA), equipped with a flame ionization detector (GC-FID). The samples were diluted in ethyl acetate (1:10) and vortexed for 60 s. Sodium sulfate was added to the samples to remove water. 1 μL of sample was injected into the gas chromatograph (1:20 split ratio) at a flow rate of 1.0 mL of helium/min. The essential oil fractions were quantified using a calibration curve containing fractions of the two standards using *n*-decane as an internal standard. The sample results were expressed: (i) in g/L with the aid of the calibration curve and (ii) in volatile percentages (%) obtained by the analysis software that provides the peak curve area, as well as the percentage of each peak in relation to the total area. Thus, the percentage of volatiles was calculated as the percentage of the volatile of interest in relation to the percentage of the other peaks added together, with the exception of the solvent.

#### 2.4.2 Characterization of Orange Essential Oil by Gas Chromatography Coupled to Mass Spectrometry (GC–MS)

To determine the profile of volatile compounds in the orange essential oil, this mixture was previously diluted in ethanol (1:100) and vortexed for 60 s. A total of 0.5 mL of each sample was added to a 10 mL vial sealed with a polytetrafluoroethylene (PTFE) septum cap and placed in a thermostatic bath (Lauda Alpha RA8, Lauda-Königshofen, Germany). After 5 min, the extraction of volatile compounds was performed by using the supported solid-phase microextraction (SPME) technique (Sigma-Aldrich 57,330). A CAR/PDMS microfiber (carboxen/poly­(dimethylsiloxane) 75 μm) was used to characterize the volatiles. The exposure time of this microfiber was 3 min. The microfiber was immediately desorbed for 3 min in the gas chromatograph inlet coupled to the mass spectrometer (GC–MS, Agilent 7890 GC system and 5975 CMSD insert, Santa Clara, CA, USA). A DB-WAX column (60 m long, 0.25 mm internal diameter, and 0.25 μm film thickness) was used, while the oven temperature was initially 80 °C, maintained for 2 min, being increased at a rate of 20 °C/min until reaching 240 °C, and maintained for 2 min. The carrier gas used in both analyses was helium gas, with a flow rate of 1 mL/min. The injector temperature was 250 °C in splitless mode. The detector was kept at 250 °C in the transfer line with electron ionization at +70 eV, working in a mass range around 35–450 *m*/*z*. Finally, the identification of compounds was performed by comparison with that of the NIST08 library.

### 2.5 Mass Transfer in SFF

Due to the high volatility of the terpenes, some loss of these compounds might be expected throughout the SFF process, especially in the depressurization step. Therefore, the amounts of limonene and α-terpineol that were effectively recovered in the fractions (*F*1 and *F*2) were estimated, helping with the assessment of possible losses. These estimations were made by means of mass balance calculations taking the solubilization cell and the separator as control volume (Figure 2).

**Figure 2:**
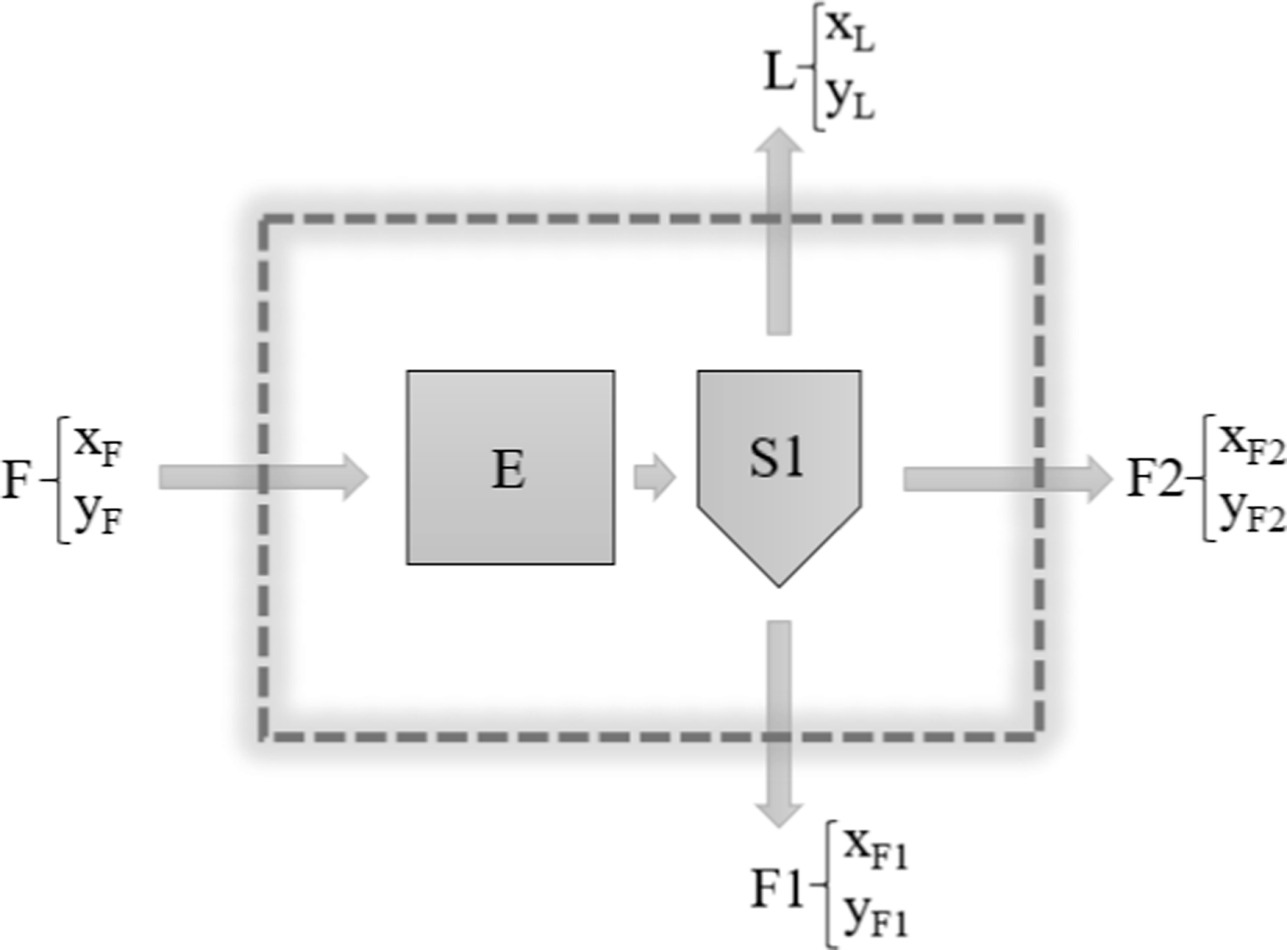
Control volume adopted for the mass balance of compounds in SFF: *E* = mass remaining in the solubilization cell; *S*1 = mass remaining in the separator; *F* = mixture of orange essential oil and α-terpineol added to the solubilization cell (10 g); *F*1 = mass fraction precipitated in the separator (measured at each SFF condition); *F*2 = mass fraction solubilized and collected in depressurization (measured at each SFF condition); *L* = volatilized mass fraction; compounds *X* and *Y* denote the masses of limonene and α-terpineol in each fraction, respectively.

Some required data for the mass balances were measured, calculated, or taken from the supplier’s information and are presented in Table 1.

**Table 1:** Information Considered for the Mass Balance of Compounds in SFF^a^

original mixture		α-terpineol	
mass (F) [g]	10.0 ± 0.1	purity [%]	96.0^a^
volume [mL]	10.1 ± 0.01	mass in the mixture [g]	4.0 ± 0.1
density [g/L]	992.1 ± 0.1	mass considering purity [g]	3.8 ± 0.1
mass of essential oil [g]	6.0 ± 0.1		

a Information provided by the supplier.

This density of the original mixture was used to estimate the volumes of fractions *F*1 and *F*2 under all process conditions that corresponded to the measured masses of these same fractions. These volumes are needed to calculate the masses of limonene and α-terpineol in the fractions using their concentrations determined by GC-FID. For commercial α-terpineol, considering the mass of 4 g used for the original mixture and the 96% purity reported on the label by the manufacturer, it was considered that the mixture placed in the solubilization cell contained 3.8 g of α-terpineol. The global and compound (limonene and α-terpineol) mass balances were formulated and solved as follows in eqs 2–7, based on the control volume shown in Figure 2. First, the global mass balance in the control volume is presented in eq 2.

2 $$M_{R}=M_{in}-M_{out}$$

where *M*_R_ = *E* + *S*1 is the total remaining mass within the control volume, *M*_in_ = *F* is the total mass entering the control volume, and *M*_out_ = *F*1 + *F*2 + *L* is the total mass leaving the control volume.

The total volatilized compounds can also be calculated under each SFF condition, as described in eq 3.

3 L=F−F1−F2

The masses of limonene in the mixture fed into the solubilization cell (*X*_*F*_) as well as in the fractions *F*1 (*X*_*F*1_) and *F*2 (*X*_*F*2_) obtained by SFF are calculated from the total mass and the limonene concentration of each stream, which was converted to mass basis (g limonene/g solution) using the density informed in Table 1. Therefore, the total amount of lost limonene (*X*_L_) was calculated with eq 4 (component mass balance from eq 3).

4 $$X_{L}=X_{F}-X_{F1}-X_{F2}$$

Finally, considering the masses of limonene that enters and leaves the control volume, the recoveries (*R*_*X*F_1__,*R*_*X*F_2__) and losses (*R*_*X*L_) of limonene (in %) were calculated using eqs 5–7.

5 $$R_{X_{F1}}=100\cdot \frac{X_{F1}}{X_{F}}$$

6 $$R_{X_{F2}}=100\cdot \frac{X_{F2}}{X_{F}}$$

7 $$R_{X_{L}}=100\cdot \frac{X_{L}}{X_{F}}$$

Similarly, the calculation procedure previously described for limonene can be applied to perform mass balance analysis for α-terpineol (*Y*). The recoveries of limonene and α-terpineol in the fractions obtained by SFF provide important information for the evaluation of the process performance.

### 2.6 Statistical Analysis

Process conditions, including pressure, temperature, and flow rate applied during solubilization and SFF, were controlled with uncertainty below 10%. The solubility and SFF experiments were performed at least in triplicate and were evaluated by analysis of variance (ANOVA) followed by Tukey’s test at α = 0.05. All statistical analyses were performed using MINITAB software (Release 16.1.0, Minitab Inc.).

## 3 Results and Discussion

### 3.1 Solubility Assessment in SC-CO_2_

Solubility data in SC-CO_2_ are of paramount importance for the separation of target compounds, such as limonene and α-terpineol in this work, as they help in selecting suitable temperature and pressure ranges for the SFF process. As previously described in Section 2.2, solubility was evaluated in the orange essential oil, α-terpineol (purity 96%) and in the mixture (that simulates a biotransformation product) of orange essential oil and α-terpineol (60:40, wt %). Since the orange essential oil is rich in limonene, knowing its solubility could support SFF results aimed at its separation. Figure 3 and Table S1 (Supporting Information) present the dynamic solubilities for each liquid system, as a function of pressure and temperature.

**Figure 3:**
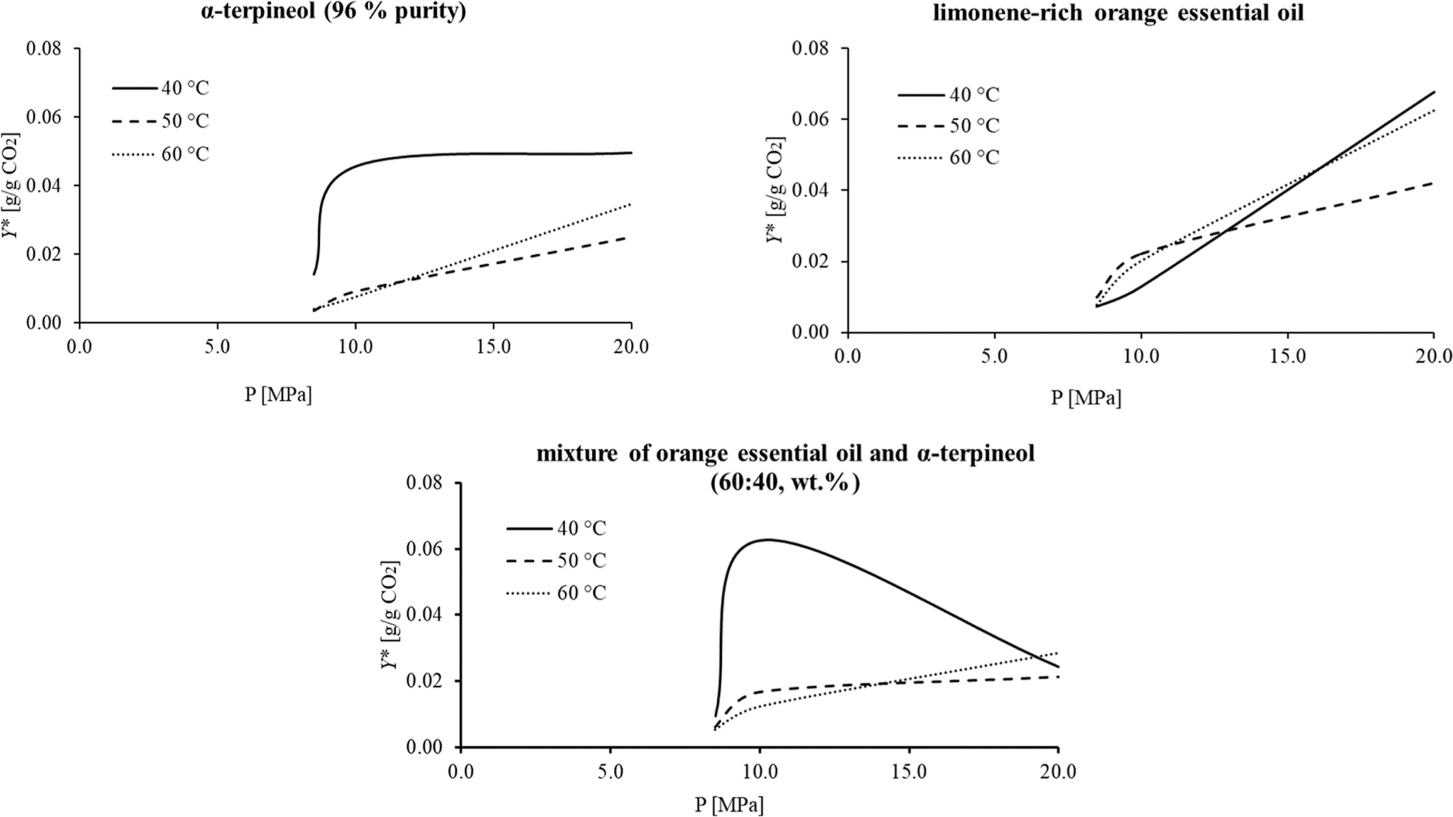
Solubilities (Y*) of α-terpineol (96% purity), limonene-rich orange essential oil, and mixture of orange essential oil and α-terpineol (60:40, wt %) in SC-CO_2_ at different temperatures and pressures.

The selected temperature and pressure ranges were based on the solubilities of orange essential oil target compounds in SC-CO_2_ reported in previous works.^14,16^ To the best of our knowledge, there are no literature data regarding the specific solubility of α-terpineol in SC-CO_2_. Therefore, this study reviewed literature on the fractionation of limonene and linalool^13,14,24^ to specify the process conditions, as linalool and α-terpineol are both oxygenated terpenes. Yet, some of these works did not evaluate pressures above 15 MPa, which leads this present work to the decision of limiting the experimental pressure up to 20 MPa.

In general, the solubilities presented in Figure 3 and Table S1 (Supporting Information) demonstrate an increase trend with pressure at a constant temperature. This is expected because the SC-CO_2_ density increases with pressure, thus enhancing solvation power. Exceptionally, the mixture at 40 °C presented the highest solubility at 10 MPa, indicating a possible crossover pressure (clearly represented when the curve reached a local maximum at a pressure of approximately 10.5 MPa in Figure 3). At this point, solute–solvent interaction is affected, and solubility could change drastically depending on the complexity of the system. In respect to the temperature, the mixture of orange essential oil and α-terpineol (60:40, wt %) presented different trends in comparison to the individual α-terpineol (96% purity) and limonene-rich orange essential oil solutions. For instance, at low pressure (8.5 MPa), increasing temperature often reduces solubility. However, when comparing the results at 20 MPa, for all systems, solubility decreased at 50 °C and turned to increase at 60 °C. This could indicate that the effects of the vapor pressure of the solutes (mixed or separated) might govern their transfer from the liquid to the supercritical phase when the CO_2_ density is high enough. The decrease in solubility with increasing temperature may be desirable for separation processes, as it enhances the selectivity of the target compound, as in this work with α-terpineol, offering a potential approach to add value to a biotransformation product. The ambiguous effect of temperature on solubility is also known as retrogradation or crossover and is often reported in the literature for vegetable and essential oils.^16,18,25^ A recent study reported by Carvalho et al.^16^ demonstrated a crossover between 13 and 15 MPa for orange peel oil in isotherms of 40, 50, and 60 °C plotted from 10 to 24 MPa. This data corroborates the findings of the dynamic solubility data of this present study, allowing us to predict that the pseudoternary mixture also presents its crossover point between nearby 15 MPa.

The molecular interactions of limonene and α-terpineol in the pseudoternary mixture (limonene + α-terpineol + CO_2_) may also have affected the solubility of these monoterpenes in SC-CO_2_. Moreover, α-terpineol has a hydroxyl group in its molecule, which increases its polarity and thus decreases its affinity with CO_2_. When observing separately the solubility behavior of α-terpineol and orange essential oil at 10 MPa and 50 °C (Table S1, Supporting Information), one can note that the solubility of α-terpineol is lower, which is expected due to the superior polarity of this compound. Moreover, the increase in the molar mass of a solute, at a given condition of temperature and pressure, tends to reduce its solubility. The molar mass of α-terpineol is 154.25 g·mol^–1^ and that of orange essential oil must be very close to that of limonene, which is 136.24 g·mol^–1^, thus, some difference in solubility could be expected. This behavior was also reported by Antonie and Pereira^15^ for some terpenes, including limonene.

Cháfer et al.^14^ also reported high pressure solubility data for the system limonene + linalool + CO_2_. They highlight the importance of ternary solubility data in order to obtain a correct thermodynamic model phase behavior of a system at high pressure, suggesting that molecular interactions between components could not be neglected. This is confirmed in this study, when comparing solubility behavior of binary (α-terpineol–SC-CO_2_) and pseudoternary systems. Based on these findings, α-terpineol could be potentially separated from orange essential oil by pressure reduction in SC-CO_2_.

The mixture of orange essential oil and α-terpineol showed its highest solubility at 10 MPa and 40 °C, within the evaluated range. Moreover, the solubility of the mixture of orange essential oil and α-terpineol decreases at 40 °C when the pressure increases from 10 to 20 MPa (Table S1, Supporting Information), reinforcing that 10 MPa and 40 °C are the most suitable conditions to solubilize it in SC-CO_2_. Therefore, this condition was selected to solubilize the mixture prior to the fractionation strategy proposed in this work, with the corresponding results discussed in the following sections of this manuscript.

### 3.2 Supercritical Fluid Fractionation (SFF)

The SFF experiments were carried out in a fractionation unit that consisted of a solubilization cell coupled to a separator, as shown in Figure 1. First, a kinetic SFF experiment was carried out in order to set a solvent-to-feed (S/F) ratio for the upcoming runs. This parameter significantly impacts the extraction efficiency and optimization, especially when there is a need for process scalability.^26^ This value represents the ratio of the supercritical solvent to the mass of the material used in the process (the mixture of orange essential oil + α-terpineol (60:40, wt %), in this work). Figure 4 presents the kinetic SFF curve, where the mass of the mixture collected in the separator was practically constant after 20 min. Based on the CO_2_ flow rate of 8.6 g/min used in this kinetic study, the calculated S/F was 17.2 g of CO_2_/g of feed. This S/F ratio was maintained for the SFF experiments at all pressures and temperatures, as described in Section 2.3.2.

**Figure 4:**
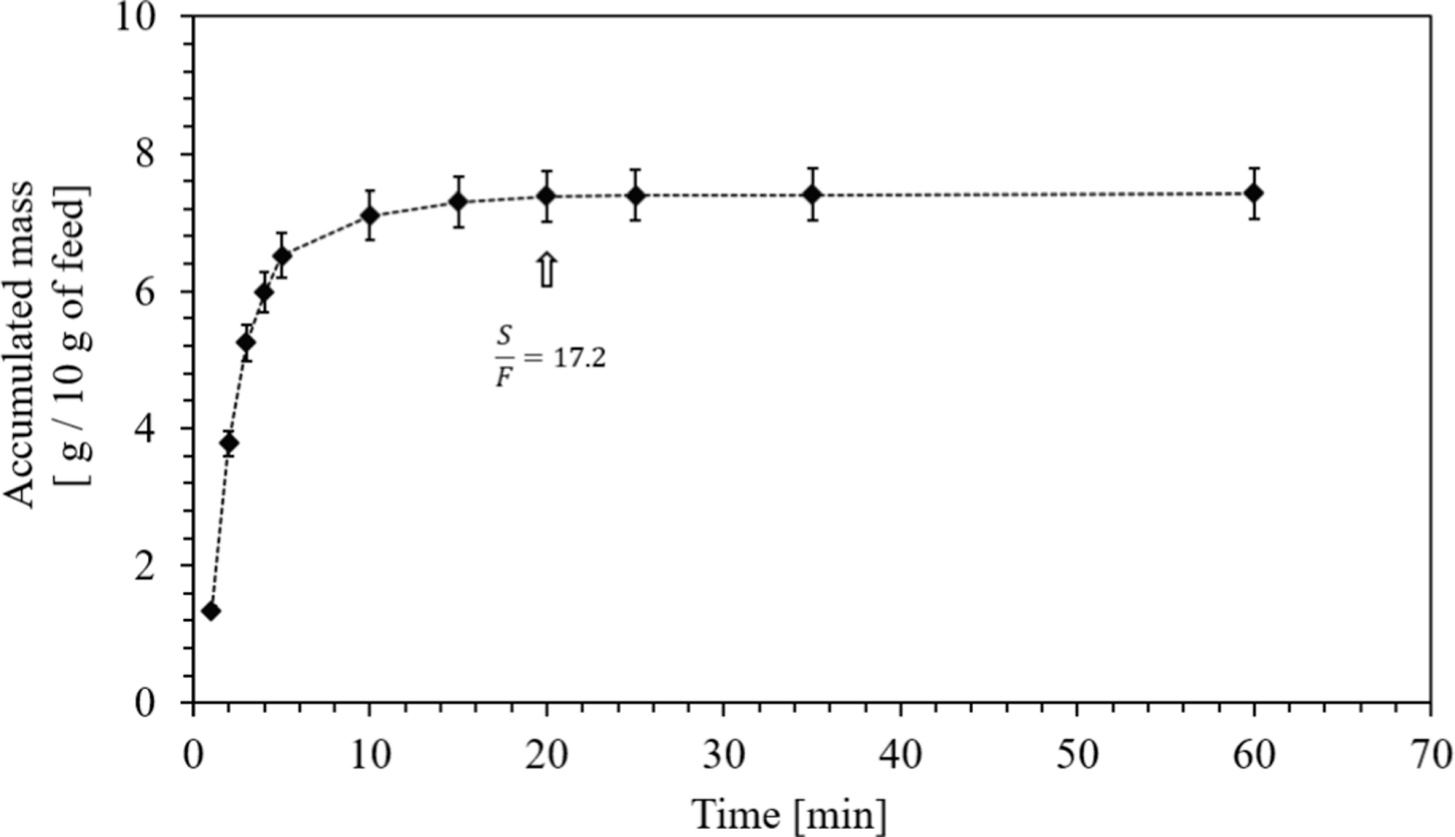
Kinetics of SFF of the mixture of orange essential oil + α-terpineol (60:40, wt %) at 7.5 MPa and 50 °C in the separator.

The mixture of orange essential oil and α-terpineol (60:40, wt %) was solubilized in supercritical CO_2_ (10.0 MPa, 40 °C) for fractionation by SFF. Subsequently, the limonene and α-terpineol contents in each fraction were determined as the percentage of volatiles quantified by GC-FID, with results summarized in Table 2. The data presented in Table 2 provide initial insights into the effect of the CO_2_ density on the solubility of both compounds and, consequently, on their separation efficiency.

**Table 2:** Percentage of Volatiles of the Mixture of Orange Essential Oil + α-Terpineol (60:40, wt %) and of the Fractions *F*1 and *F*2 Obtained by SFF under Different Conditions of Pressure (*P*) and Temperature (*T*)­^a^ ^,^ ^b^

SFF condition			limonene [%]		α-terpineol [%]	
*T* [°C]	*P* [MPa]	CO_2_ density [kg/m^3^]^a^	*F*1	*F*2	*F*1	*F*2
40	6.0	149.26	59.14 ± 0.37^DE^	73.12 ± 0.31^BC^	32.81 ± 0.02^A^	20.82 ± 0.26^B^
50		135.21	59.89 ± 1.25^D^	80.62 ± 0.94^A^	32.11 ± 0.76^A^	13.93 ± 0.88^C^
60		124.91	58.51 ± 1.70^DE^	79.61 ± 1.34^A^	32.63 ± 1.10^A^	14.81 ± 3.40^C^
40	7.0	198.02	59.41 ± 0.33^D^	76.36 ± 0.01^ABC^	32.72 ± 0.30^A^	17.76 ± 0.01^BC^
50		172.01	58.33 ± 0.43^DE^	80.42 ± 1.78^A^	33.41 ± 1.25^A^	14.00 ± 1.65^C^
60		155.53	58.39 ± 0.33^DE^	80.40 ± 1.60^A^	33.71 ± 0.31^A^	14.02 ± 1.47^C^
40	8.0	277.90	54.50 ± 2.45^E^	71.77 ± 1.37^C^	36.81 ± 2.40^A^	21.94 ± 1.28^B^
50		219.18	57.01 ± 2.41^DE^	77.24 ± 0.94^AB^	34.88 ± 2.23^A^	16.74 ± 0.87^BC^
60		191.62	56.33 ± 2.94^DE^	76.59 ± 2.65^ABC^	35.59 ± 2.70^A^	17.31 ± 2.37^BC^
mixture essential oil + α-terpineol			61.82 ± 0.13		30.50 ± 0.13	

a Values estimated from the NIST WebBook; means with different letters in the same column differ statistically by Tukey’s test (α = 0.05); *F*1: fraction collected in the separator; *F*2: fraction collected after depressurization.

b Mixture prior fractionation solubilized at 10 MPa and 40 °C.

Under all temperature and pressure conditions in the SFF process, it can be observed that the percentage of α-terpineol in *F*1 was higher than that in the original mixture, ranging from 32.11 to 36.81%, although these values were not statistically different, suggesting that these CO_2_ densities were equally efficient to promote the concentration of α-terpineol, even at the tested densities (from 124.91 to 277.90 kg/m^3^). Interestingly, it is noticed that despite the mass fraction of α-terpineol representing 40% of the mixture, only 30.50% of the compound was accounted among the total volatile content as quantified by gas chromatography, possibly due to the high volatility of this compound and difficulties associated with its manipulation.

Moreover, the percentage of limonene in *F*1 was lower than in the original mixture for all the applied conditions. This result indicates SFF’s capability to concentrate α-terpineol from the original mixture, even though a more efficient condition was not identified among those tested in this study. A recent work reported by García-Fajardo et al.^17^ aimed at a nonthermal separation process for deterpenation of orange essential oil. The authors verified the effect of temperature and pressure (vacuum) on a molecular distillation process, reducing the limonene content in the deterpenated fraction to 47.96%, and concentrating linalool (also an oxygenated terpene as α-terpineol) up to 8.3-fold, compared to the initial concentration in the feed prior distillation at 1.5 mmHg and 35 °C. However, when increasing the vacuum to the pressure of 2 mmHg at the same temperature, the authors did not find an effective separation, yielding similar limonene content in both distillate and deterpenated fraction. These findings suggest small variations in pressure, under the same temperature applied, could make the separation of limonene from other oxygenated terpenes difficult.

In general, a remarkable percentage of limonene is also found in *F*1 (even though it is still lower than in the original mixture), while α-terpineol is slightly higher concentrated, indicating a limited selectivity of fractionation under the investigated conditions of CO_2_ density. This may indicate that in this condition, the solubility of α-terpineol in SC-CO_2_ is lower than in the other conditions evaluated, while limonene remains more soluble so that some separation is achieved.

It is important to emphasize that, although higher temperatures could be evaluated, the compounds present in the essential oil and in the mixture are thermolabile.^17,27^ Thus, the studied temperature range was carefully chosen to prevent their degradation.

Besides the solubility of compounds in the tested CO_2_ densities, the chemical nature of the target compounds (limonene and α-terpineol) and other components in the mixture to be fractionated could also have affected the process. The complex composition of orange essential oils, which do not include only limonene (orange essential oil composition is presented in Table 3), besides the waxes of high molecular weight previously identified by Carvalho et al.,^28^ hinders the selective precipitation of pure α-terpineol, which is an oxygenated hydrocarbon. Therefore, this system could face limitations from solvent and the vapor pressure of all its components combined.

**Table 3:** GC–MS Chemical Composition of the Orange Essential Oil Donated by Citrosuco^a^

identified compound	relative peak area [%]
heptane	1.60
cyclopropane	1.03
cyclohexane	1.69
α-pinene	1.01
β-pinene	2.73
*R*-limonene	76.21
β-terpinyl-acetate	1.74

a The values in Table 3 correspond to the relative peak areas obtained by GC–MS analysis, expressed as the percentage of each compound with respect to the total area of identified volatile peaks (excluding the solvent). Therefore, these values represent a relative distribution of the volatiles and are not based on the absolute mass of the collected fraction.

The chemical composition analysis confirmed the predominance of limonene in the orange essential oil used in this study. This content was lower than that reported by García-Fajardo et al.^17^ who found 92.6% limonene in orange essential oil. Their work also characterized a byproduct from a different citrus processing company, highlighting the variability in composition that can arise from differences in fruit variety or local cultivation practices. It is also noteworthy that the target compound of this work (i.e., α-terpineol) was not detected in the composition of this orange essential oil, reinforcing the need to create a model mixture to simulate a biotransformation product that would be more concentrated in α-terpineol.

Figure 4 presents the concentrations of limonene and α-terpineol in the precipitated (*F*1) and depressurized (*F*2) fractions obtained by SFF after mixture solubilization in SC-CO_2_. The concentrations of both compounds in the mixture are also informed to support comparison and discussion.

According to Figure 5, the concentration of α-terpineol in the precipitated fraction (*F*1) was higher than in the mixture under all SFF conditions, while the limonene concentration is reduced, corroborating the observations found for the respective volatile percentages in this fraction (Table 2). These results reinforce the ability of the SFF process to concentrate α-terpineol under the evaluated conditions, although the phase behavior in SC-CO_2_ should be deeply investigated. Also, in line with these observations, the best scenario for the effective separation was found at the highest CO_2_ density of 277.9 kg/m^3^, in which the concentration of α-terpineol slightly exceeded limonene’s. Finally, regarding *F*2, all conditions concentrated limonene by a larger extent than α-terpineol in the mixture.

**Figure 5:**
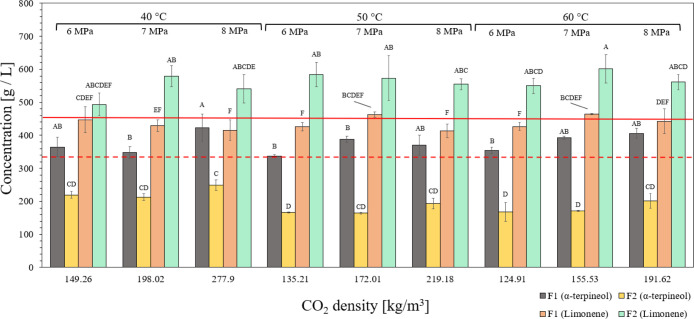
Concentrations of α-terpineol and limonene fractions obtained by SFF from the mixture of orange essential oil and α-terpineol (60:40, wt %) at different temperatures and pressures. *F*1: fraction collected in the separator. *F*2: fraction collected at depressurization. Different letters indicate significant differences statistically evaluated by Tukey’s test (α = 0.05). The compounds in the mixture (prior fractionation) revealed limonene at 457 g/L and an α-terpineol concentration of 334 g/L, which are represented by red straight and dashed lines, respectively.

The current challenge is to find an ideal condition in which α-terpineol prevails over limonene in the separator (*F*1), with the remaining compounds remaining soluble in SC-CO_2_ and recovered at depressurization (*F*2).

The results presented in Table 2 and Figure 5 confirm that selectivities of the compounds limonene and α-terpineol in SC-CO_2_ are strictly dependent on molecular interactions rather than on CO_2_ density. For instance, Cháfer et al.^14^ demonstrated, in a ternary system composed of limonene:linalool (60:40, wt %) and SC-CO_2_, that effective separation was achieved, resulting in a limonene to linalool mass ratio of almost 4, at 70 bar and 45 °C. It is important to highlight, however, that the authors worked with a pure ternary system, in which conditions would be far from a real orange oil system model as that used in this work, which explains the challenge in the effective separation in the same.

### 3.3 SFF Mass Balance

In order to account for the total mass of the compounds aimed for separation in this study (with high volatility), mass balance calculations were performed. Although GC-FID results were expressed in g/L, all values were converted into absolute masses and recoveries (%), ensuring that process efficiency is evaluated on a mass basis. The GC-FID analyses clearly revealed the presence of limonene and α-terpineol in the two fractions obtained (*F*1 and *F*2) in SFF, as discussed in the previous sections. The calculated amounts of these compounds in each fraction are depicted in Table 4.

**Table 4:** Calculated Masses and Recoveries (%) of Limonene and α-Terpineol Obtained in the Separator (*F*1) and after Depressurization (*F*2) by SFF from a Mixture of 10 g of Orange Essential Oil and α-Terpineol (60:40, wt %) Solubilized at 10 MPa and 40 °C^a^

SFF condition			limonene				α-terpineol		
*T* [°C]	*P* [MPa]	*X*_*F*1_ [g]	*R*_*X*_*F*1__ (%)	*X*_*F*2_ [g]	*R*_*X*_*F*2__ (%)	*Y*_*F*1_ [g]	*R*_*Y*_*F*1__ (%)	*Y*_*F*2_ [g]	*R*_*Y*_*F*2__ (%)
40	6.0	3.91	65.20	0.09	1.40	3.19	83.00	0.04	1.00
50		3.68	61.30	0.12	2.00	2.91	75.80	0.03	0.90
60		3.46	57.70	0.15	2.50	2.88	74.90	0.05	1.20
40	7.0	3.52	58.70	0.26	4.30	2.86	74.40	0.09	2.50
50		3.52	58.70	0.21	3.60	2.95	76.80	0.06	1.60
60		3.26	54.30	0.29	4.90	2.75	71.60	0.08	2.20
40	8.0	2.63	43.80	0.39	6.40	2.67	69.50	0.18	4.60
50		3.00	50.00	0.52	8.60	2.69	70.10	0.18	4.70
60		3.27	54.40	0.65	10.9	3.00	78.00	0.23	6.10

a *X*_*F*1_ and *X*_*F*2_: masses of limonene in *F*1 and *F*2, respectively; *Y*_*F*1_ and *Y*_*F*2_: masses of α-terpineol in *F*1 and *F*2, respectively; *R*_*X*_*F*1__ and *R*_*X*_*S*2__: recoveries of limonene in *F*1 and *F*2, respectively; *R*_*Y*_*F*1__ and *R*_*Y*_*F*2__: recoveries of α-terpineol in *F*1 and *F*2, respectively.

It can be clearly noted that the percent recoveries of α-terpineol in the separator (*R*_*Y*_*F*1__) were higher than those that remained solubilized (*R*_*Y*_*S*2__) and were collected after depressurization. This confirms that most of the α-terpineol contained in the initial mixture effectively precipitated under all of the SFF conditions.

However, for limonene, recoveries in F_1_ were lower than those of α-terpineol. Additionally, the sums of the recoveries for limonene and α-terpineol in both *F*1 and *F*2 did not achieve 100% under any condition, indicating that part of the mixture subjected to SFF could have been lost by volatilization in depressurization or remained inside the solubilization cell or in the separator, thus not being recovered and accounted for. That suggests that high pressure systems, even if the temperature is controlled, might offer some drawbacks when the aim is to concentrate compounds with high volatility such as terpenes.

The mass balance approach also allowed calculating the total loss of the mixture (eq 4), which accounts for both volatilized and precipitated material. Moreover, the percent losses of limonene and α-terpineol were also calculated from the component mass balances. Table 5 shows the losses calculated through the global and component mass balances.

**Table 5:** Calculated Mass Losses in the SFF Process Obtained from Global and Component Mass Balances^a^

SFF condition		total loss	Limonene		α-terpineol	
*T* [°C]	*P* [MPa]	*L* [g]	*X*_L_ [g]	*R*_*X*_L__ (%)	*Y*_L_ [g]	*R*_*Y*_L__ (%)
40	6.0	1.16	2.00	33.39	0.61	15.88
50		1.23	2.20	36.68	0.89	23.17
60		1.68	2.39	39.83	0.92	23.95
40	7.0	1.42	2.22	37.03	0.89	23.17
50		2.08	2.27	37.77	0.83	21.61
60		2.57	2.45	40.85	1.01	26.30
40	8.0	3.02	2.99	49.76	0.99	25.75
50		1.86	2.48	41.35	0.97	25.26
60		1.53	2.08	34.70	0.61	15.88

a *L*: total mass loss during the SFF process; *X*_L_: mass of limonene lost during SFF; *R*_*X*_L__: percent loss of limonene; *Y*_L_: mass of α-terpineol lost during SFF; *R*_*Y*_L__: percent loss of α-terpineol.

As observed in the results presented in Table 5, there were considerable losses at all SFF conditions, mainly for limonene (>2 g) over a total of 10.0 g of mixture fed into the system. It is particularly notable that the highest percent losses of limonene and α-terpineol (*R*_XTL_ and *R*_YTL_), highly volatile compounds, occurred at 7.0 MPa and 60 °C, and at 8.0 MPa and 40 °C, respectively, indicating losses are likely to be associated with process arrangements rather than selectivity. Despite the remarkable amount of unrecovered α-terpineol, SFF at 8.0 MPa and 40 °C achieved the highest concentrations of α-terpineol in a recovered fraction (Figure 5). Therefore, the SFF process could be enhanced by preventing precipitation in the solubilization cell and controlling the collection of the precipitated material. Possibilities to achieve these scenarios include improved depressurization control, the use of multistage separators, or cryogenic collection traps for high volatile compounds, which could be effective strategies to improve recovery in future process designs.

In addition, the orange essential oil used in the mixture may contain other compounds with higher molecular mass, such as sesquiterpenes and diterpenes, waxes that consist of fatty acids and phospholipids^28^ and others, that were not quantified by GC-FID. These compounds must also have precipitated since their higher molecular mass and polarity make them much less soluble in CO_2_ than limonene and α-terpineol, thus altering the molecular interaction and selectivity of the process.

Some hypotheses can be raised to explain the low degree of separation between limonene and α-terpineol and the higher losses during SFF: (i) limonene is soluble in SC-CO_2_ and, therefore, the highest amounts of limonene in *F*2 are found at the highest evaluated pressures. However, the solubility of limonene may not be stable under the evaluated process conditions. For instance, in a pioneer investigation on phase equilibrium reporting limonene and SC-CO_2_, Matos et al.^29^ described a high affinity of limonene with SC-CO_2_ above 9.8 MPa, and below this pressure, the solubility tended to decrease. This may explain the fact that limonene had its solubility reduced and, thus, a great part of it precipitated in the separator together with α-terpineol under the SFF conditions performed in this work; (ii) the static solubilization time of 15 min for the mixture may not have been long enough to saturate the SC-CO_2_, so part of the limonene could not be effectively dissolved when entering the separator, which resulted in its precipitation. In this case, a longer contact time between the solvent and the mixture would be necessary to achieve complete solubilization and subsequent fractionation. Therefore, longer static times should be tested.

In the search for a more efficient separation process between limonene and α-terpineol, a good understanding of the thermodynamic equilibrium in extremely complex systems such as essential oils and mixtures enriched with pseudocompounds may be needed. It is necessary to comprehend in more detail the behavior of these two components in complex systems to identify the differences between their interactions with CO_2_ and with other components of orange essential oil, in order to develop a more effective separation of α-terpineol, avoiding the simultaneous precipitation of limonene or other compounds.

## 4 Conclusions

A model mixture containing orange essential oil and α-terpineol was used to simulate a product resulting from the biotransformation of limonene, with the aim to separate α-terpineol by SFF using SC-CO_2_. The best solubilization of the mixture was achieved at 10 MPa and 40 °C. The SFF process was capable of precipitating most of the α-terpineol from the mixture containing orange essential oil and α-terpineol (60:40, wt %), and a CO_2_ density of 277.9 kg/m^3^ (8 MPa and 40 °C) was the most promising condition to achieve such separation. However, under the evaluated SFF conditions, the process was not completely efficient in separating α-terpineol and limonene since most of the limonene also precipitated in the separator, evidencing a complex multicomponent system where molecular interactions could play an important role in selectivity. Therefore, a wider range of CO_2_ densities should be investigated, as well as other mixture compositions, to better understand the interactions of α-terpineol and limonene in SC-CO_2_ and, thus, seek a more efficient separation process. In this aspect, it is also important to deepen the thermodynamic knowledge of the system under the applied conditions and under other conditions. For this, experimental and theoretical studies of phase equilibrium for systems containing orange essential oil (or limonene) + α-terpineol + CO_2_ are recommended.

It is also possible that the chemical natures of α-terpineol and limonene are not distant enough to provide very different solubilities in SC-CO_2_ under the investigated SFF conditions. In this sense, further work should explore a broader range of CO_2_ densities, alternative mixture compositions, and phase equilibrium data to improve the separation efficiency. Complementary separation techniques may also be required to enhance α-terpineol purification.

## Supplementary Material



## Notes

Solubilities (*Y**) of limonene-rich orange essential oil, α-terpineol (96% purity), and mixture of orange essential oil and α-terpineol (60:40, wt %) in SC-CO_2_ at different temperatures and pressures (PDF)
