# Supplementary material for: Separation of α‑Terpineol and Limonene from an Orange Essential Oil Mixture Using Supercritical CO2 Pressure Reduction
Source: ACS Omega. 2025 Oct 25;10(43):51912–22. doi: 10.1021/acsomega.5c08436 (PMC12593098; doi:10.1021/acsomega.5c08436)
Supplement: Supplementary file 1 [file ao5c08436_si_001.pdf]

Separation of α-terpineol and limonene from orange essential oil mixture using supercritical CO_2 pressure reduction

Rayanne Priscilla França de Melo[a], Rafael Chelala Moreira[b], Glaucia Maria Pastore[b], Juliano Lemos Bicas[b], Julian Martínez[a], Luana Cristina dos Santos[a,c*]

[a]University of Campinas, School of Food Engineering, Department of Food Engineering and Technology, 80, Monteiro Lobato Street, 13083-862 Campinas, São Paulo, Brazil.

[b]University of Campinas, School of Food Engineering, Department of Food Science and Nutrition, 80, Monteiro Lobato Street, 13083-862 Campinas, São Paulo, Brazil.

[c]Food Science Research Institute (CIAL), CSIC-UAM, Nicolás Cabrera 9, 28049 Madrid, Spain.

[*]Corresponding author: e-mail address: *luana.dsantos@csic.es*

Supplementary material

Table S1. Solubilities (*Y**) of limonene-rich orange essential oil, α-terpineol (96 % purity), and mixture of orange essential oil and α-terpineol (60:40, wt.%) in SC-CO_2 at different temperatures and pressures.

Compounds	P [MPa]	*Y** [g/g CO_2]		
		**40 °C**	**50 °C**	**60 °C**
Limonene-rich orange essential oil	8.5	0.007±0.001^{dCD†}	0.009±0.001^{deB}	0.008±0.001^{efCD}
	10.0	0.012±0.001^{cdDEF}	0.0221±0.0001^{bcC}	0.020±0.001^{dCD}
	20.0	0.068±0.002^{aA}	0.042±0.007^{aBC}	0.063±0.005^{aA}
α-terpineol	8.5	0.014±0.002^{cdA}	0.0035±0.0004^{eG}	0.0039±0.0004^{fFG}
	10.0	0.046±0.003^{bB}	0.0091±0.0004^{deEF}	0.008±0.001^{efF}
	20.0	0.049±0.005^{abAB}	0.025±0.003^{bDE}	0.035±0.004^{bBCD}
Mixture	8.5	0.0093±0.0004^{dBC}	0.0061±0.0004^{eDE}	0.0053±0.0002^{fEF}
	10.0	0.062±0.006^{aA}	0.017±0.002^{cdDE}	0.012±0.001^{eEF}
	20.0	0.024±0.002^{cDE}	0.021±0.001^{bcE}	0.028±0.002^{cCDE}

†Means and standard deviation with different lowercase letters differ isothermally by Tukey's test and uppercase letters differ isobarically by Tukey's test (α = 0.05).
